# Identification of Anoikis‐Related Genes in Gastric Cancer: Bioinformatics and Experimental Validation

**DOI:** 10.1002/cam4.70907

**Published:** 2025-04-22

**Authors:** Chao Song, Wenbo Liu, Xiaoyu Wang, Xin Liu, Zhiran Yang, Yingying Wang, Qun Zhao, Yong Li, Mingming Zhang, Bibo Tan

**Affiliations:** ^1^ The Third Department of Surgery The Fourth Hospital of Hebei Medical University Shijiazhuang China; ^2^ Department of Anesthesiology Hebei General Hospital Shijiazhuang China; ^3^ Hebei Key Laboratory of Metabolic Diseases/Clinical Medicine Research Center Hebei General Hospital Hebei China; ^4^ Graduate School Hebei Medical University Shijiazhuang China

**Keywords:** anoikis, consensus clustering, CYP1B1, experimental validation, gastric cancer

## Abstract

**Introduction:**

Distant metastasis is the main reason for the poor prognosis of gastric cancer, and anoikis refers to the cell death caused when cells detach from the extracellular matrix or adhere in incorrect locations, playing an important role in the distant metastasis of gastric cancer.

**Methods:**

Download the TCGA‐STAD dataset and the anoikis gene set, and filter out the differentially expressed anoikis genes. Perform consensus clustering of gastric cancer samples, and conduct Weighted Gene Correlation Network Analysis (WGCNA), enrichment analysis, and immune infiltration analysis for the expression characteristics of each subtype, while also filtering the genes with differential expression between subtypes. Additionally, through COX survival analysis, identify anoikis genes related to gastric cancer prognosis and establish a nomogram. Finally, validate the differentially expressed gene CYP1B1 in vivo and in vitro through clinical samples, cell culture, and the establishment of an anoikis model.

**Results:**

Three subtypes of gastric cancer with anoikis genes were identified, each exhibiting different expression characteristics, biological pathways, and immune cell infiltration. The abundance of activated NK cells, memory B cells, and M2 macrophages showed significant differences among the three subtypes. We screened four differentially expressed gene sets and five genes (CYP1B1, EQTN, NRXN2, TBC1D3E, TCEAL5) among the three subtypes. Through survival analysis, we identified 33 independent prognostic genes and constructed a nomogram, with calibration curves indicating good consistency. Finally, we selected CYP1B1 for experimental validation, and in vivo and in vitro experiments demonstrated that CYP1B1 is highly expressed in gastric cancer, participates in the resistance to cell death in gastric cancer cells, and promotes the invasion, migration, and tumor progression of gastric cancer cells.

**Conclusion:**

The expression patterns of subtypes based on differentially expressed genes related to anoikis in gastric cancer vary, providing theoretical support for the future of personalized treatment for gastric cancer.

**Abbreviations**
ARGs: anoikis‐related genes
BP: biological process
CC: cellular component
DEGs: differentially expressed genes
ECM: extracellular matrix
GC: gastric cancer
GO: gene ontology
GPCR: G protein‐coupled receptor
GSEA: gene set enrichment analysis
ICD: immunogenic cell death
KEGG: kyoto encyclopedia of genes and genomes
MF: molecular function
NICR: national infrastructure of cell line resource
ssGSEA: single sample gene set enrichment analysis
STAD: stomach adenocarcinoma
WGCNA: weighted gene correlation network analysis

## 1 Introduction

Gastric cancer (GC) is the fifth most common cancer and the third most common cause of death from cancer worldwide, seriously endangering people's health [1]. According to the Global Cancer Report 2020, there are more than 760,000 deaths and 1 million new patients worldwide each year [2]. Due to the lack of specific symptoms in the early stage, patients with gastric cancer are usually diagnosed at an advanced stage, characterized by lymph node and distant metastasis [3, 4, 5]. Currently, surgical treatment is the main treatment method, but for advanced patients, the loss of surgical opportunity due to distant metastasis is one of the main reasons for their low survival rate [6, 7]. Increasing evidence suggests that changes in genes and corresponding biological processes play a significant role in the process of tumor metastasis [3, 4]. Therefore, studying the expression of these genes is of great significance for controlling tumor metastasis.

Anoikis is a special type of programmed cell death that occurs when adherent cells detach from the extracellular matrix (ECM) [8, 9, 10, 11]. Anoikis was first discovered in endothelial cells and adherent cells, closely related to the stability of the intracellular environment [12, 13]. Currently, studies have shown that anoikis acts as a significant barrier to tumor metastasis [11], which prevents tumor cells from being planted in inappropriate places [12, 13]. However, Under pathological conditions, tumor cells that acquire malignant potential develop the ability to survive and grow in non‐anchored conditions, known as anoikis resistance. Tumor cells that gain anoikis resistance are closely related to cancer metastasis in patients and represent a key step in the process of tumor metastasis [14, 15]. The presence of anoikis‐resistant tumor cells is an important reason for the survival and colonization of circulating tumor cells during the metastatic process [9, 16, 17]. Several studies have shown that Anoikis‐Related Genes(ARGs) play an important role in the development of cancer [18]. Understanding the anoikis resistance ability of gastric cancer cells is crucial for developing methods to prevent cancer cells from metastasizing to distant organs [19, 20, 21, 22, 23].

In this study, we conducted a comprehensive and in‐depth analysis of gastric cancer subtypes classified based on anoikis genes. A number of studies have delineated subtypes of lung adenocarcinoma [23], cutaneous melanoma [24] and hepatocellular carcinoma [25] based on ARGs [26], but little has been done in the field of gastric cancer. We screened for anoikis genes that are differentially expressed in normal and gastric cancer tissues, performed consensus clustering of cancer samples, and conducted a study of the different subtypes by WGCNA, enrichment analysis, immune infiltration analysis, prognostic analysis, and differential analysis. We conducted in vivo and in vitro experimental validation of the differential gene CYP1B1. Current clinical treatment strategies mainly rely on TNM staging, but this is not sufficient to predict prognosis because highly complex genetic heterogeneity can cause patients with the same clinical symptoms and pathological stages to show different prognoses [27], so we established a nomogram based on the differentially expressed genes(DEGs) between different subtypes, which provides a theoretical basis for personalized treatment of gastric cancer patients.

## 2 Materials and Methods

### 2.1 Data Collection

RNA‐seq expression data of stomach adenocarcinoma (STAD) and patients' clinical information were downloaded from the TCGA database (https://portal.gdc.cancer.gov/), and we obtained 375 cancer samples and 32 normal samples; the collection of anoikis‐associated genes was obtained from the GeneCards database (https://www.genecards.org/).

### 2.2 Screening for Anoikis‐Related Differential Genes

The “Limma” package in R language was used to normalize the data and screen for DEGs. The Wilcoxon rank sum test was used to perform non‐parametric tests on the data. |Fold Change| > 1.5 and False Discovery Rate(FDR) < 0.05 were used to screen up and down regulated ARGs in gastric cancer samples. The “Pheatmap” package and “ggplot” package in R language were used to plot heatmaps and volcano maps of DEGs. Pearson’ correlation was applied to calculate differential gene expression correlations and miRNA‐mRNA regulatory relationships were downloaded in Starbase (http://starbase.sysu.edu.cn/).

### 2.3 Single Sample Gene Set Enrichment Analysis

Single sample gene set enrichment analysis (ssGSEA) is an extension of the gene set enrichment analysis (GSEA) method that calculates enrichment scores for each sample and gene set pair. Each ssGSEA enrichment score represents the extent to which a specific gene set member is up or down regulated in the sample. Consensus clustering of cancer samples was performed by calculating the ssGSEA score of differentially expressed ARGs for each gastric cancer sample.

### 2.4 Consensus Clustering Analysis

Consensus clustering analysis was performed on the cancer samples using the “ConsensusClusterPlus” package in R language to further investigate the role and prognostic importance of ARGs in STAD, and the Calinski‐Harabaz index was used as an evaluating index for the clustering of data to determine the optimal k‐value of the clusters. It was obtained from the ratio of separation to tightness, with separation representing the degree of dispersion between subgroups and tightness representing the degree of aggregation within categories, and the larger the ratio indicates the better the clustering effect. The “Survival” package in R language was used for survival analysis, and *p* < 0.05 was considered to be statistically significant.

### 2.5 Weighted Gene Correlation Network Analysis

The “WGCNA” package in R language was used to construct co‐expression networks for each subtype. Firstly, the samples of each subtype were hierarchically clustered to detect and eliminate outliers; secondly, the pickSoft Threshold function was used to build a scale‐free topology network for each subtype; thirdly, the adjacency matrix was constructed and transformed into a TOM matrix, the co‐expression network was constructed based on the optimal soft threshold, and the gene clustering tree was drawn after dividing the genes into different modules; finally, a correlation heatmap between genes and a correlation heatmap between modules and clinical features, *p* < 0.05 was considered statistically significant.

### 2.6 Enrichment Analysis

The “Metascape” (https://metascape.org) was used for the enrichment analysis and visualization of Gene Ontology (GO) and Kyoto Encyclopedia of Genes and genomes (KEGG) pathways for each subtype. Reactome Database (http://reactom.org/) was used for the analysis of reactome pathways for each subtype. GO is a common method for gene function enrichment, which covers three main categories: molecular function (MF), cellular component (CC), and biological process (BP). KEGG is a database that integrates genomic, chemical, and systemic functional information and is mainly used for analyses of biometabolic pathways. The Reactome database is similar to the KEGG database in that it brings together the reactions and biological pathways of human biological processes.

### 2.7 Immune Cell Infiltration Analysis

The “Cibersort” package in R language was used to calculate the immune cell infiltration level of each subtype after consensus clustering. The “Pheatmap” package in R language was used to draw heat maps. In addition, the degree of enrichment of ARGs within each sample and the enrichment score within each immune cell were calculated by ssGSEA, respectively. Spearman's correlation was used to calculate the correlation between the pooled scoring of differentially expressed anoikis genes and the immune cell infiltration level.

### 2.8 Screening for Hub‐Genes

Using ANOVA to identify genes that are differentially expressed in different subtypes, FDR < 0.05 was considered statistically significant; enrichment analysis of differentially expressed genes was performed based on “Metascape”. The “limma” package in R language was applied to screen the differentially expressed genes of each subtype. The criteria of |Fold Change| > 1.5 and FDR < 0.05 were followed. “Pheatmap” package and “ggplot” package were used to draw heatmaps and volcano maps of differentially expressed genes.

### 2.9 Prognostic Analysis and Construction of Nomogram

Independent prognostic genes were screened by “Survival” package by performing univariate and multivariate COX regression survival analyses for genes differentially expressed in at least two subtypes, and *p* < 0.05 was considered statistically significant. The “rms” package in R language was applied to integrate the independent prognostic genes, and a nomogram was constructed to predict the 3–5‐year survival of the patients, and the calibration graph was used to verify the degree of agreement between the model and the actual situation.

### 2.10 Clinical Samples

Three pairs of fresh gastric cancer tissues, adjacent non‐tumor tissues, and liver metastatic tissues were used for qRT‐PCR and partially Western Blotting. All samples were collected from January 2022 to September 2022 at the Fourth Hospital of Hebei Medical University, with the three pairs of sample providers being two males and one female, all aged between 60 and 65 (Table S1). The diagnosis of each gastric cancer patient was confirmed by two independent pathologists, and none of the patients underwent radiotherapy, chemotherapy, or immunotherapy before the biopsy. This study was approved by the Ethics Committee of the Fourth Hospital of Hebei Medical University (Project No: 2019ME0039), and written informed consent was obtained from each patient before sample collection. Our research was conducted in accordance with the Declaration of Helsinki.

### 2.11 Cell Culture

Normal gastric mucosal epithelial cells GES‐1 and gastric cancer cell lines (MKN45, AGS, HGC‐27) were purchased from the National Infrastructure of Cell Line Resource (NICR). The cells were cultured in DMEM medium (Gibco, USA) containing 10% fetal bovine serum, 100 U/mL penicillin, and 100 μg/mL streptomycin at a temperature of 37°C with 5% CO_2_ and humidity.

### 2.12 Construction of Anoikis Model

After resuspending the adherent cells with trypsin, seed the cells at a density of 1 × 10^6^ cells per well in a 6‐well plate with ultra‐low attachment. Then, place them in a 37°C, 5% CO_2_ incubator for culture. After 24 h of incubation, proceed with the subsequent experiments.

### 2.13 qRT‐PCR

According to the kit instructions, total RNA was extracted from gastric cancer cells and gastric cancer tissues using TRIzol reagent (Invitrogen, USA). Total RNA was reverse transcribed using the PrimeScriptTM RT Reagent Kit (TaKaRa, Japan). qRT‐PCR was performed using the Quantstudio DX system (Applied Biosystems, Singapore). β‐actin was used as the internal reference gene, and the relative expression levels were calculated using the $2^{-\Delta \Delta C_{T}}$ method. Primer sequences are listed in Table S4.

### 2.14 Western Blot

GC cells and tissues were homogenized by adding lysate and Protease Inhibitor Cocktail and centrifuged at 12,000 rpm at 4°C for 5 min, and the supernatant was collected. The protein concentration in the supernatant was detected by the BCA kit. Proteins were separated by SDS‐polyacrylamide gel electrophoresis and transferred to a PVDF membrane. This was followed by incubation with a primary antibody overnight at 4°C, and the membrane was washed with TBST and incubated with a labeled secondary antibody at room temperature for 2 h. The following primary antibodies against CYP1B1 (Abcam, ab185954), N‐Cadherin (Abcam, ab76011), E‐Cadherin (Abcam, ab314063), Vimentin (Abcam, ab92547), Snail (Abcam, ab216347), ITGAV (Abcam, ab179475), CL‐caspase‐7 (Abcam, ab256469), CL‐caspase‐3 (Abcam, ab32042), Bcl‐2 (Abcam, ab182858), Bax (Abcam, ab32503), β‐actin (Abcam, ab8227) were used. The signal was visualized using enhanced chemiluminescence (ECL) (Thermo Fisher, USA). The ratio of the target protein to the internal reference protein was calculated to derive the relative expression of the proteins.

### 2.15 Transwell Assay and Wound Healing Assay

The cell invasion ability was measured using 24‐well Transwell chambers with 8.0‐μm pore polycarbonate filters (CoStar, USA). 1 × 10^5^ cells were seeded into Matrigel‐coated chambers. The upper chambers contained 200 μL of serum‐free medium, and the lower chambers contained 500 μL of complete medium. After 24 h, the cells across the membrane were fixed with 4% paraformaldehyde and stained with 0.2% crystal violet for 20 min to identify the invaded cells. The number of stained cells was counted in three random fields. For the wound healing assay, cells were seeded in a 6‐well plate at a density of 2 × 10^4^ per well, cultured until 80%–90% confluence, and scratched with a 1000 μL pipette tip. Cells were rinsed with PBS, and the medium was replaced with serum‐free medium at 37°C for 24 h. The artificial wound was assessed at 0 h and 24 h post‐scratching. The distance for the migration was measured by Image J.

### 2.16 Cell Transfection

To construct the CYP1B1 knockdown plasmid, shRNA targeting CYP1B1 (sh‐CYP1B1 group) and negative control shRNA (sh‐Ctrl group) were synthesized and cloned into the pCDH‐CMV‐MCS‐EF1‐copGFR plasmid, and GC cells were transfected with the plasmid according to the manufacturer's instructions. The target sequences of shRNAs are shown in Table S2.

### 2.17 Animal Models

All animal experiments were approved by the Committee on the Ethics of Animal Experiments of Hebei Medical University. Six weeks old male balb‐c nude mice were purchased from Beijing Hufukang Biotechnology Co., LTD., and randomly divided into knockdown control group (sh‐Ctrl group) and CYP1B1 knockdown group (sh‐CYP1B1 group), with 5 mice in each group, to evaluate the effect of CYP1B1 on mice. 2 × 10^6^ MKN45 cells were injected into the right side of the mice. Tumor growth was monitored every 3 days, and the volume was calculated using the formula 0.5 × length × width^2^. After 24 days, the mice were euthanized through intravenous injection of sodium pentobarbital, following the “Laboratory animal—Guidelines for euthanasia”, and the tumors were removed for further analysis.

## 3 Results

### 3.1 Identification of Differentially Expressed Genes

**FIGURE 1:**
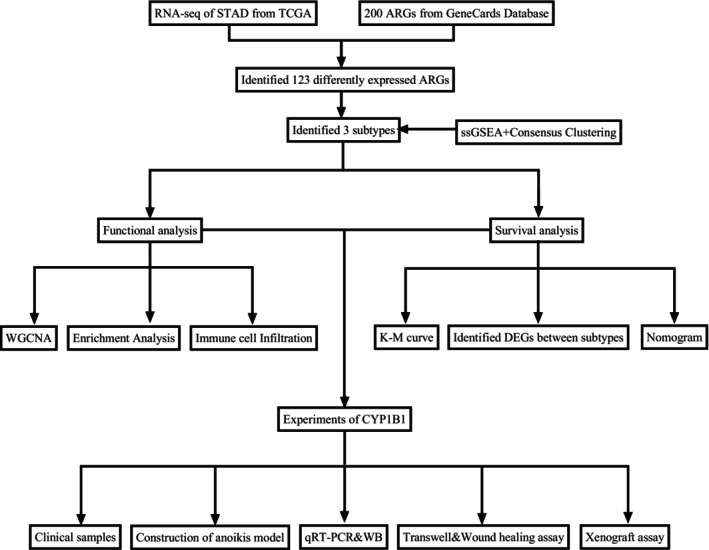
Flowchart of the study.

**FIGURE 2:**
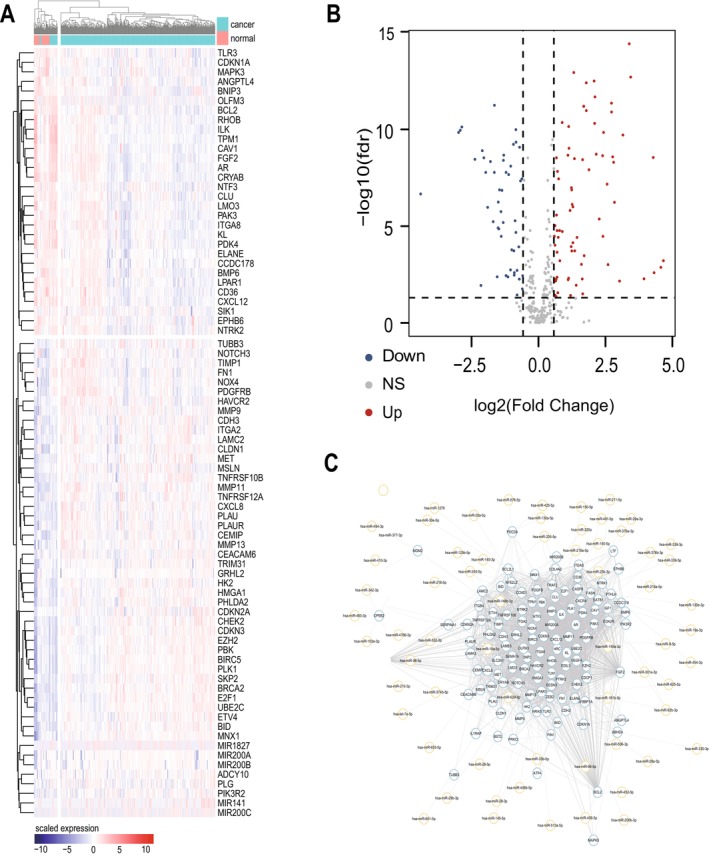
Identification of differentially expressed genes. (A) Heatmap of differentially expressed ARGs between gastric cancer tissues and normal tissues. (B) Volcano plot of differentially expressed ARGs between gastric cancer tissues and normal tissues. (C) Expression network of differentially expressed ARGs.

The flow chart of the study is shown in Figure 1. To evaluate the expression patterns of ARGs in gastric cancer, clinical information and RNA‐seq expression data were obtained from the TCGA database in 375 gastric cancer patients and 32 normal subjects. 338 ARGs were obtained from the GeneCards database. We found that a total of 333 genes were differentially expressed between normal tissues and gastric cancer tissues (Table S5), among which 74 genes were up‐regulated and 49 were down‐regulated. Only the DEGs of |Fold Change| > 2 and FDR < 0.01 were shown in this paper. We used the “Pheatmap” package to generate heat maps of differential genes through hierarchical clustering, in which red represents up‐regulated gene expression and blue represents down‐regulated gene expression (Figure 2A). “ggplot2” package is used to generate the volcano map; the horizontal coordinate represents Log2 (Fold Change), and the vertical coordinate represents −Log10(False Discovery Rate); each point represents a gene; red represents the up‐regulated differential gene, blue represents the down‐regulated differential gene, and black represents genes that are not significantly differentially expressed (Figure 2B). At the same time, we constructed the differential gene expression network and queried the miRNA expression regulation corresponding to each differential gene (Figure 2C).

### 3.2 Identification of Gene Subtypes Related to Anoikis in Gastric Cancer

**FIGURE 3:**
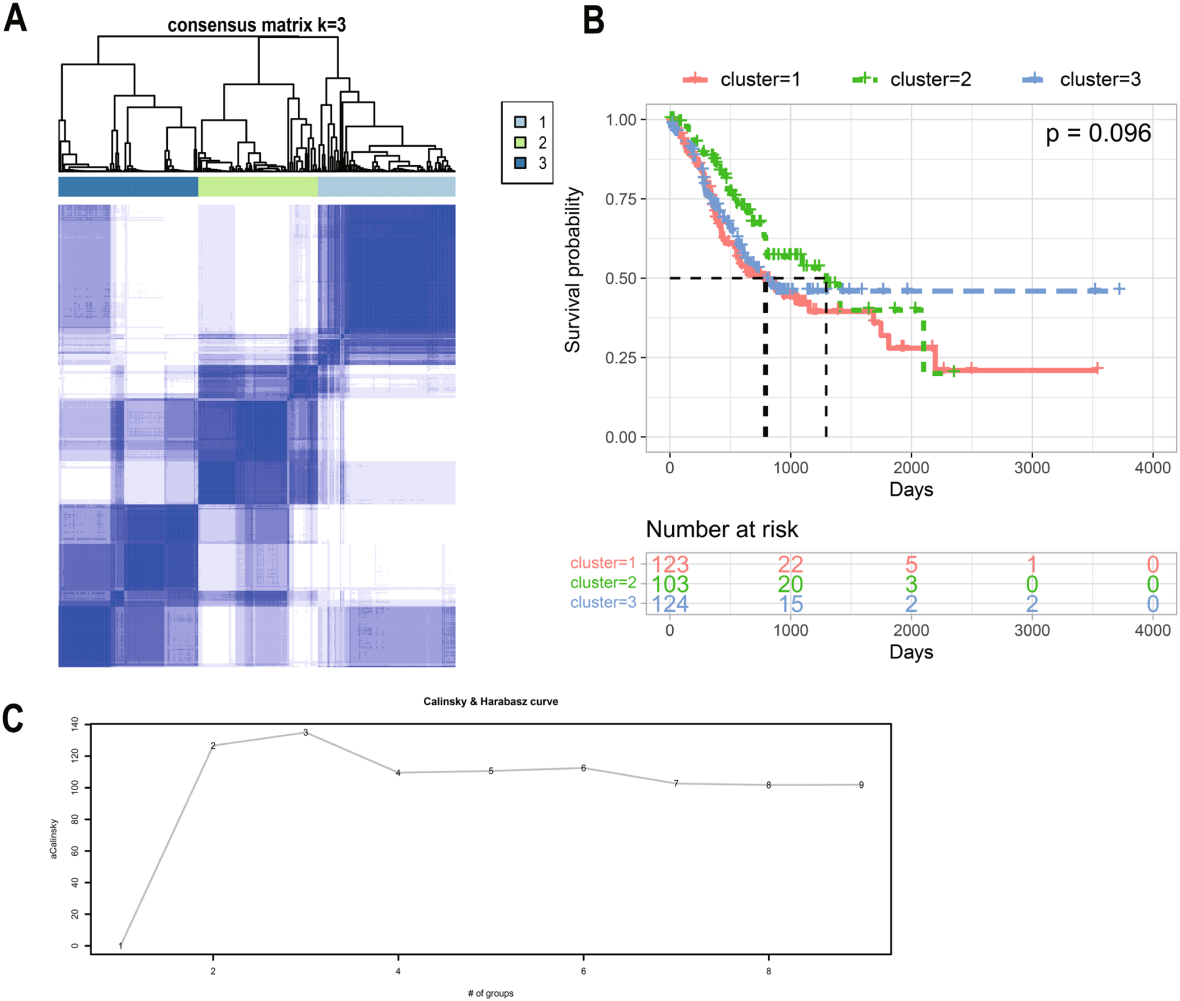
Gene subtypes related to anoikis in gastric cancer. (A) Subgrouping of gastric cancer samples using consensus clustering. (B) Survival analysis of the three subgroups. (C) *K* = 3 is the optimal number of groups.

In order to better study the expression characteristics of ARGs in gastric cancer, we calculated the ssGSEA score of differentially expressed ARGs in each sample and categorized the gastric cancer samples by consensus clustering method (Figure 3A). We evaluated *k* = 3 as the best number of groups by using the Calinski‐Harabaz index (Figure 3C). The horizontal coordinate represents the number of groups, and the vertical coordinate represents the evaluation index. The larger the corresponding ratio of the vertical coordinate, the smaller the covariance of the data within the categories, the larger the covariance between the categories, and the better the clustering effect. K‐M analysis showed no significant difference in survival time between the three subtypes(*p* = 0.096) (Figure 3B).

### 3.3 Weighted Gene Correlation Network Analysis

**FIGURE 4:**
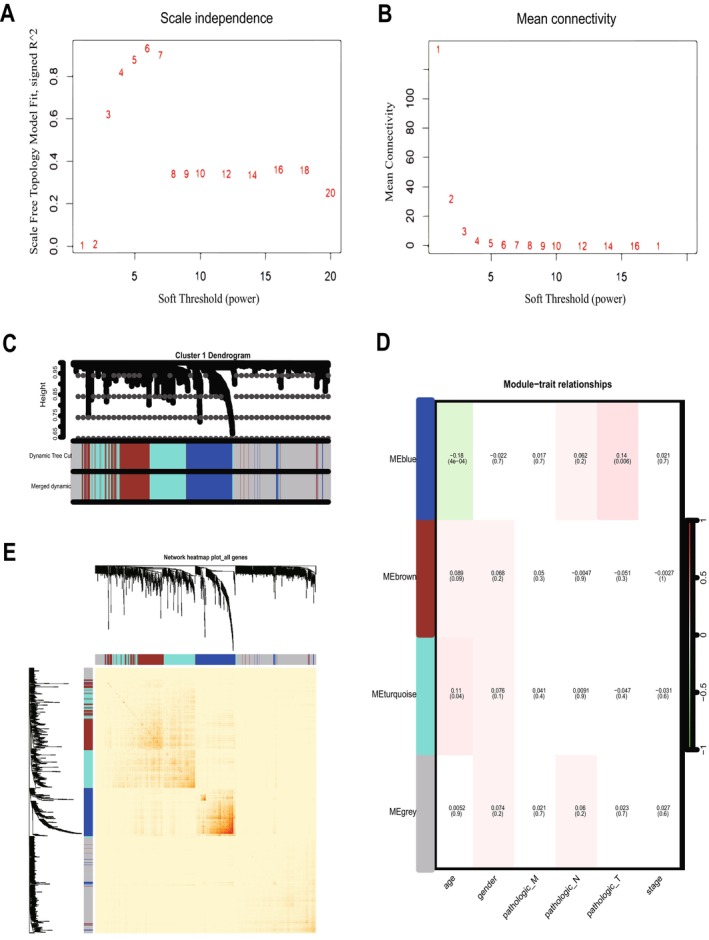
WGCNA of cluster1. (A, B) According to the optimal soft threshold β, cluster1 is divided into 4 modules. (C) Gene clustering map of cluster1. (D) Heatmap of the correlation between modules and clinical traits in cluster1. (E) The correlation heatmap of the genes in cluster1.

**FIGURE 5:**
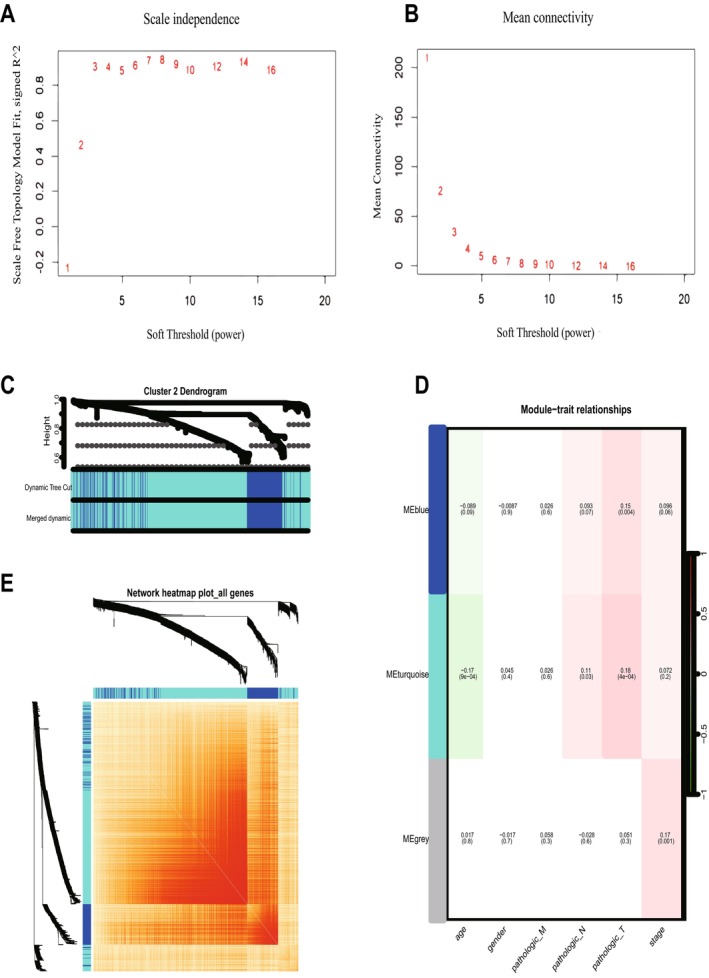
WGCNA of cluster2. (A, B) According to the optimal soft threshold β, cluster1 is divided into 3 modules. (C) Gene clustering map of cluster2. (D) Heatmap of the correlation between modules and clinical traits in cluster2. (E) The correlation heatmap of the genes in cluster2.

**FIGURE 6:**
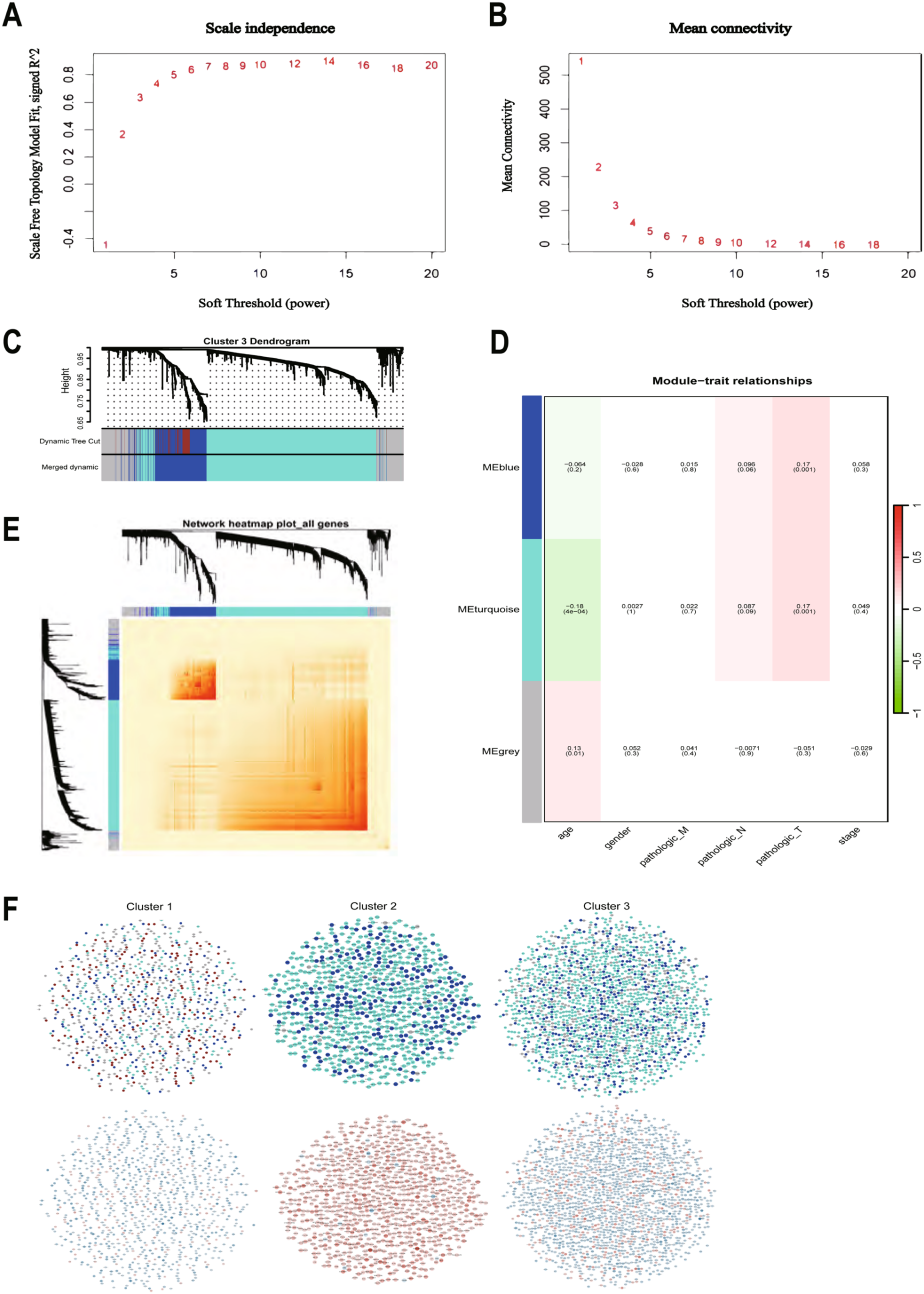
WGCNA of cluster3. (A, B) According to the optimal soft threshold β, cluster1 is divided into 3 modules. (C) Gene clustering map of cluster3. (D) Heatmap of the correlation between modules and clinical traits in cluster3. (E) The correlation heatmap of the genes in cluster3. (F) Expression changes of each cluster gene.

To determine the relationship between each subtype and clinical features, we performed WGCNA for each of the three subtypes. The optimal soft threshold β for cluster1, cluster2, and cluster3 was calculated to be 5, 3, and 7, respectively. Based on the optimal soft threshold β, cluster1, cluster2, and cluster3 were classified into 4, 3, and 3 modules, respectively. Among them, the genes in the gray module are not clustered into any module, so they belong to the invalid module. In cluster1, we calculated the scale‐free fit index under different soft thresholds, and we chose 0.8 as the value criterion, so the minimum soft threshold of cluster1 is 5 (Figure 4A), and we also calculated the average connectivity under different soft thresholds (Figure 4B). Firstly, we plotted the gene clustering map (Figure 4C); the upper part is the tree diagram of hierarchical clustering of genes, and the lower part is the gene module, which corresponds to the top and bottom. Secondly, we plotted the correlation heatmap of the genes; the darker the color, the stronger the interactions between genes, and since the diagonal line indicates the interactions between genes within the module, the darkest color is found on the diagonal line (Figure 4E). Finally, we plotted a heatmap of the correlation between modules and clinical traits within each subtype; the darker the color the higher the correlation, red represents positive correlation, green represents negative correlation, and the number above each cell represents correlation while the number below represents significance (Figure 4D), in which the blue module was significantly correlated with clinical T stage(*p* = 0.006), while the correlation with age was the poorest (*p* = 0.0004). Meanwhile, we determined the optimal soft threshold β in cluster2 and cluster3, respectively, and plotted gene clustering, correlation heatmap between genes, and clinical trait correlation heatmap. The turquoise module were significantly correlated with clinical T stage in cluster2 (*p* = 0.0004) (Figure 5D). Both the blue module and the turquoise module were significantly correlated with clinical T stage in cluster3 (*p* = 0.001) (Figure 6D), while the turquoise module was the least correlated with age in cluster2 (*p* = 0.0009) (Figure 5D) and cluster3 (*p* = 0.0004) (Figure 6D).

And we showed the expression changes of each cluster gene (Figure 6F). The upper layer is the WGCNA clustering results; each point represents a gene, and the color of each point corresponds to the color within the module. We can visualize the number of genes in each cluster and the number of genes contained in the module. The lower layer is the gene expression changes; we can visualize the up‐regulation and down‐regulation of genes within each cluster. Red indicates up‐regulation of gene expression, green indicates gene expression down‐regulation; the darker the color, the higher the degree of gene up‐regulation or down‐regulation.

### 3.4 Enrichment Analysis and Immune Infiltration

**FIGURE 7:**
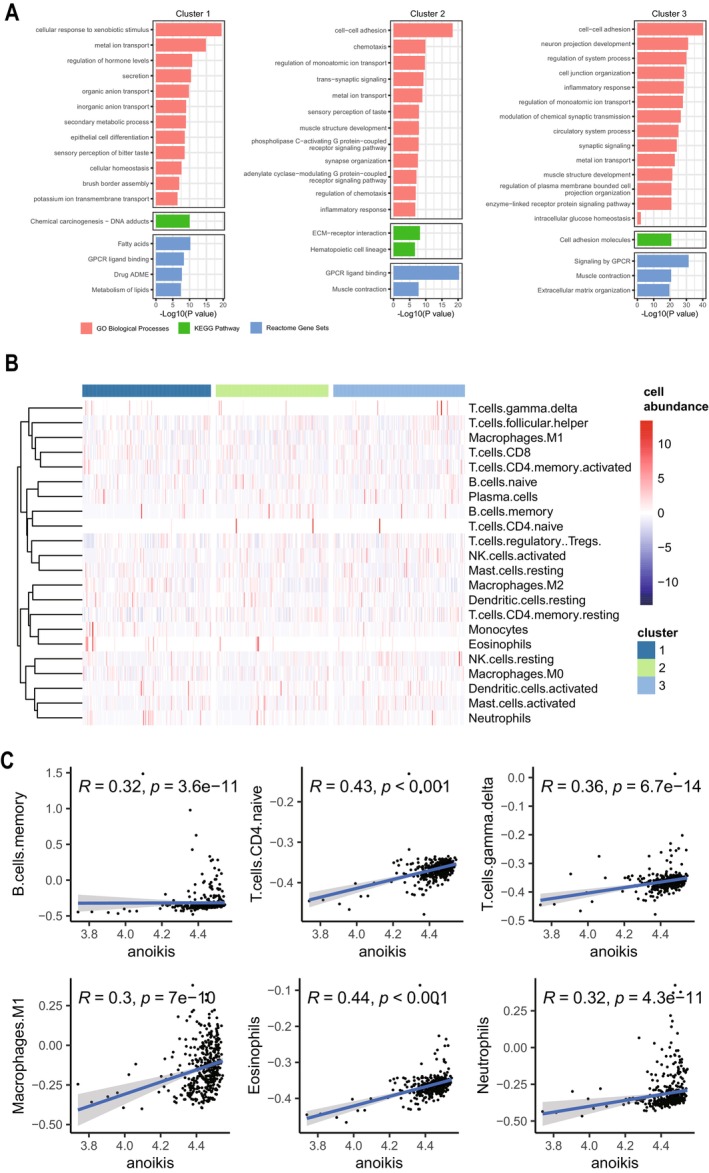
Enrichment analysis and Immune infiltration of 3 subtypes. (A) Enrichment analysis of GO, KEGG, and Reactom. (B) Heatmap of immune cells. (C) Correlation between immune cells and anoikis scoring.

GO and KEGG enrichment analyses showed that cluster1 was significantly enriched in response to xenobiotic stimulus, chemical carcinogenesis; cluster 2 and cluster 3 were significantly enriched in cell–cell adhesion, extracellular matrix (Figure 7A). Reactom enrichment analyses showed that cluster1 was enriched in the fatty acids signaling pathway, cluster 2, and cluster 3 were both significantly enriched in the G protein‐coupled receptor(GPCR) signaling pathway (Figure 7A). We found that the gene function‐enriched regions of cluster1 were different from those of cluster 2 and cluster 3, and the gene function‐enriched regions of cluster 2 and cluster 3 were almost the same, which was also consistent with the results of the analysis of WGCNA. Cluster 2 and cluster 3 were mainly clustered on intercellular adhesion and extracellular matrix, which was consistent with the apoptotic properties of anoikis, which is also closely related to the clinical staging. The immune cell infiltration levels of the three subtypes were calculated separately by the “Cibersort” package, and the results showed that the abundance of activated NK cells (*p* = 0.021), memory B cells (*p* = 0.025), and M2 macrophages (*p* = 0.039) differed significantly among the three subtypes, with a *p* < 0.05 (Figure 7B) (Table S3). The correlation between ssGSEA scores and immune cell infiltration levels was calculated by the Cibersort, spearman's correlation, and the results showed that except for memory B cells (*p* = 3.6e‐11), T cells CD4 naive (*p* < 0.001), T cells gamma delta (*p* = 6.7e‐14), M1 macrophages (*p* = 7e‐10), eosinophils (*p* < 0.001), and neutrophils (*p* = 4.3e‐11) were all positively correlated with the ARGs, and the correlation coefficients, R, were all greater than 0.3, showing moderate correlation (Figure 7C), and *p* was less than 0.05, which was statistically significant. In conclusion, these results suggest that ARGs can provide insight into the immune response and immune infiltration of GC.

### 3.5 Screening for Differential Genes Between Different Subtypes

**FIGURE 8:**
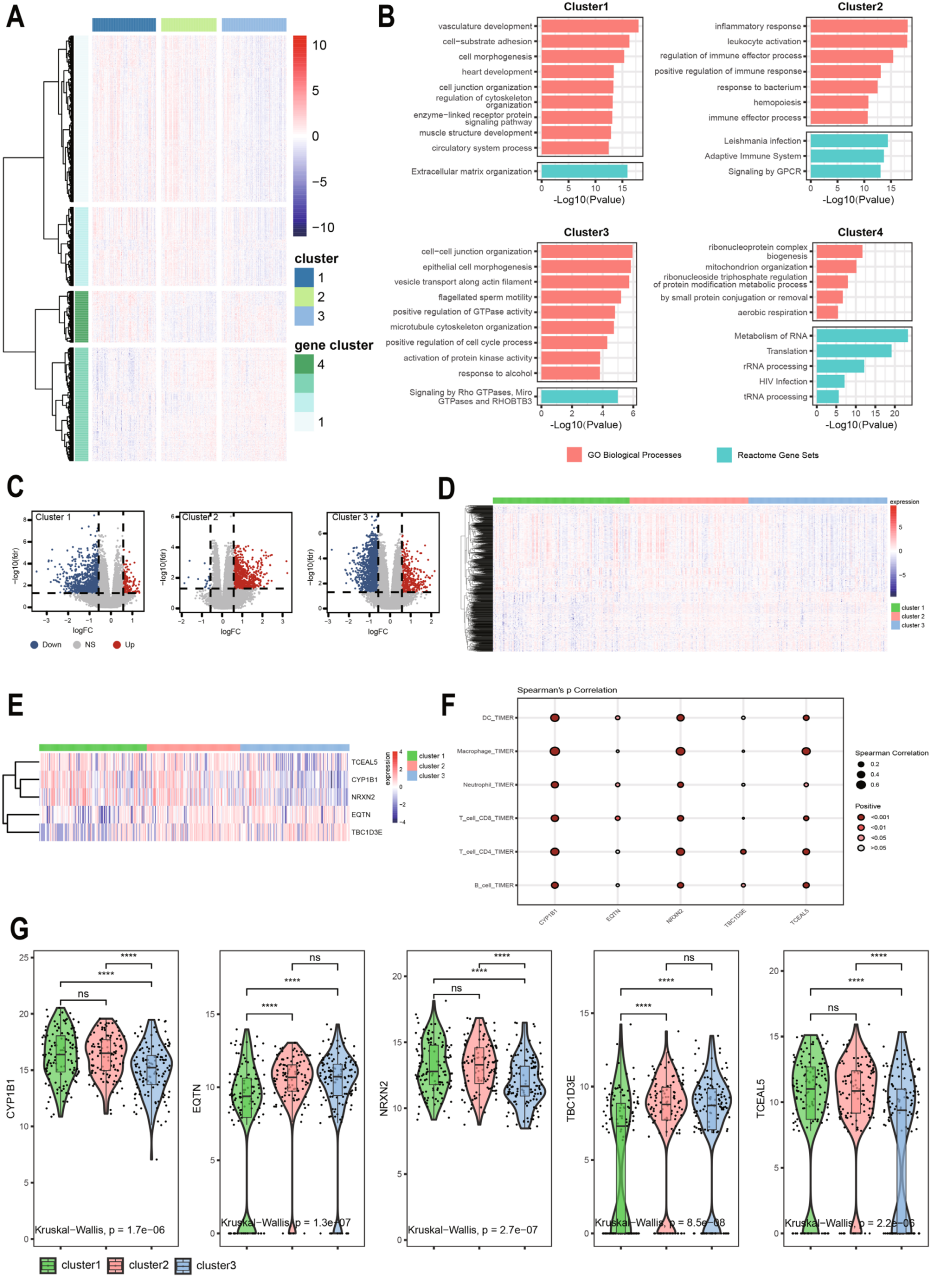
Screening for differentially expressed genes between 3 subtypes. (A) Heatmap between 3 subtypes and 4 gene clusters. (B) Enrichment analysis of 4 gene clusters. (C) Volcano maps of 3 subtypes of DEGs. (D) Heatmap of differentially expressed genes in at least two subtypes. (E, G) Five differentially expressed genes between all three subtypes. (F) TIMER analysis of CYP1B1, EQTN, NRXN2, TBC1D3E, TCEAL5. ****p < 0.0001.

In order to more comprehensively study the expression of differentially expressed genes among the three different subtypes, we analyzed each subtype. By ANOVA we identified four sets of genes that were differentially expressed in different subtypes (Figure 8A), and enriched each of the four sets of genes (Figure 8B). We found that cluster1 and cluster3 were both enriched in cell adhesion. At the same time, we screened for differential genes between the three subtypes, and when differential genes were identified for one of the subtypes, genes from the other two subtypes served as controls. |Fold Change| > 1.5 and FDR < 0.05 were used as screening criteria. Eventually, we identified 983, 849, and 1877 differentially expressed genes in cluster1, cluster2, and cluster3, respectively (Figure 8C). Among them, 917 genes were differentially expressed in at least two subtypes (Figure 8D); five genes were differentially expressed between all three subtypes (*p* < 0.05) (Figure 8E,G), namely CYP1B1, EQTN, NRXN2, TBC1D3E, and TCEAL5. Among them, CYP1B1, NRXN2, and TCEAL5 were found in gene cluster1; EQTN and TBC1D3E are in gene cluster3. Finally, we have performed TIMER algorithm analysis on 5 genes that were differentially expressed among the three subtypes (Figure 8F). We have found that among the five genes, CYP1B1 shows the strongest correlation and the most significant statistical significance (*p* < 0.001), providing a theoretical basis for future research.

### 3.6 Survival Analysis and Establishment of Nomogram

**FIGURE 9:**
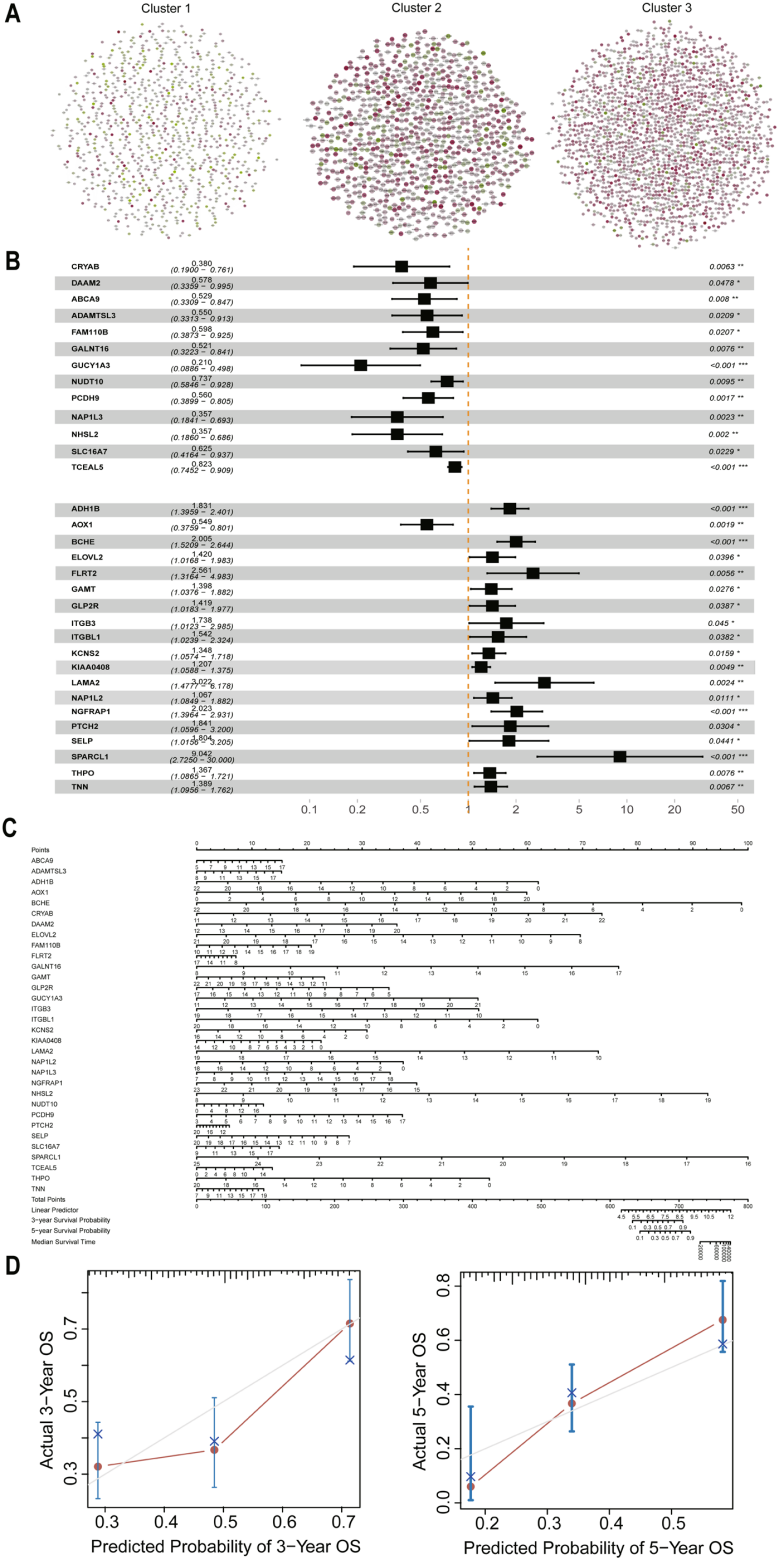
Establishment of nomogram between 32 DEGs. (A) Survival analyses in the three subtypes. (B) Multivariate COX regression of 917 differentially expressed genes in at least two subtypes. (C) Establishment of nomogram between 32 DEGs. (D) Calibration curve of nomogram.

Survival analyses were performed for all genes in the three subtypes (Figure 9A), with green dots representing HR < 1, unfavorable for survival; and red dots representing HR > 1, favorable for survival. The “Survival” package in R language was used to screen out all genes with a significance of *p* < 0.05 in univariate cox regression and differentially expressed in at least two clusters, and multivariate cox regression analyses were performed to screen for independent prognostic factors (Figure 9B). Finally, we screened 32 prognostically relevant genes (ABCA9, ADAMTSL3, ADH1B, AOX1, BCHE, CRYAB, DAAM2, ELOVL2, FAM110B, FLRT2, GALNT16, GAMT, GLP2R, GUCY1A3, ITGB3, ITGBL1, KCNS2, KIAA0408, LAMA2, NAP1L2, NAP1L3, NGFRAP1, NHSL2, NUDT10, PCDH9, PTCH2, SELP, SLC16A7, SPARCL1, TCEAL5, THPO, TNN). We found that TCEAL5 was differentially expressed among the three subtypes and was an independent prognostic factor, which implies that TCEAL5 is likely to be a new target for gastric cancer treatment. A nomogram was constructed by applying 32 independent prognostic factors (Figure 9C), and the calibration graph showed that the prognosis predicted by the nomogram model was in high agreement with the actual situation (Figure 9D).

### 3.7 CYP1B1 Promotes the Progression of Gastric Cancer In Vivo and In Vitro

**FIGURE 10:**
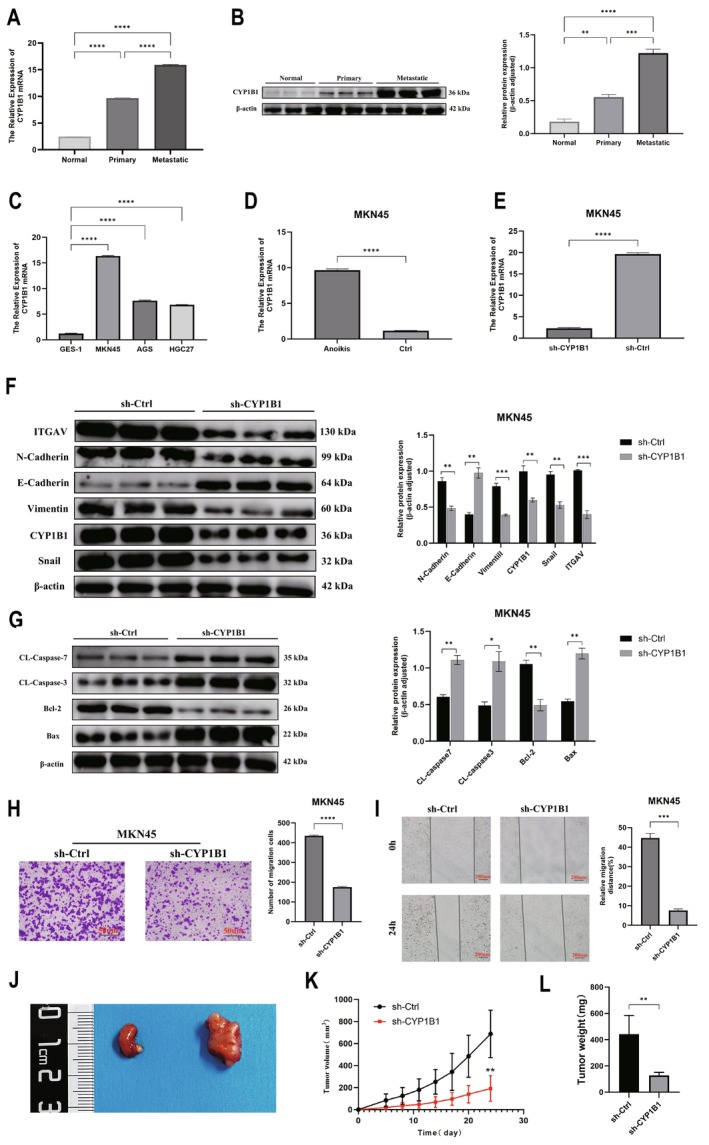
Experimental validation of CYP1B1 in gastric cancer. (A, B) The results of qRT‐PCR and WB indicate that the expression of CYP1B1 increases sequentially in normal tissue, gastric cancer tissue, and liver metastatic tissue in patients with gastric cancer liver metastasis. (C) CYP1B1 expression is highest in MKN45 gastric cancer cells. (D) CYP1B1 expression increases in a suspended state. (E) The CYP1B1 low expression group has lower CYP1B1 expression than the control group. (F, G) In the sh‐CYP1B1 group, E‐Cadherin is highly expressed, while CYP1B1, ITGAV, N‐Cadherin, Vimentin, and Snail proteins are lowly expressed. At the same time, the expression of the anti‐apoptotic protein Bcl‐2 decreases, while the expression of pro‐apoptotic proteins CL‐caspase3, CL‐caspase7, and Bax increases. (H, I) CYP1B1 promotes the invasion and migration of gastric cancer cells. (J–L) CYP1B1 promotes tumor growth in mice. *p < 0.05, **p < 0.01, ***p < 0.001, ****p < 0.0001.

We chose CYP1B1 for experimental validation. Clinical tissue samples of gastric cancer liver metastasis were selected, and the results of qRT‐PCR (Figure 10A) and WB (Figure 10B) showed that the expression of CYP1B1 increased sequentially in normal tissue, gastric cancer tissue, and liver metastatic tissue. To analyze the impact of CYP1B1 on the malignant behavior of gastric cancer, we quantified its expression in normal gastric cells (GES‐1) and GC cell lines (MKN45, AGS, HGC27), with the highest expression of CYP1B1 observed in MKN45 cells (Figure 10C). For modeling the anoikis of gastric cancer cells, gastric cancer cells were cultured in ultra‐low attachment plates for 24 h, and the qRT‐PCR results showed that CYP1B1 expression was higher than in the non‐suspended group (Figure 10D). By constructing CYP1B1 low‐expression cells and control groups through lentiviral transfection, after suspension culture, the qRT‐PCR results indicated that the CYP1B1 low‐expression group had lower CYP1B1 expression than the control group (Figure 10E, Figure S1). WB results showed high expression of E‐Cadherin and low expression of CYP1B1, ITGAV, N‐Cadherin, Vimentin, and Snail proteins, indicating a non‐EMT tendency (Figure 10F). Meanwhile, the expression of the anti‐apoptotic protein Bcl‐2 decreased in the CYP1B1 low‐expression group, while the pro‐apoptotic proteins CL‐caspase3, CL‐caspase7, and Bax increased (Figure 10G). Finally, the effect of CYP1B1 expression on cell invasion and migration was analyzed through Transwell and scratch assays. The results indicated that low expression of CYP1B1 inhibited the migration of gastric cancer cells (Figure 10H,I). To further verify the in vitro experimental results, sh‐Ctrl MKN45 cells and sh‐CYP1B1 MKN45 cells were subcutaneously injected to establish a subcutaneous tumor model in nude mice (Figure 10J). We observed changes in the volume of subcutaneous tumors and plotted the corresponding growth curves, with results showing that CYP1B1 knockdown significantly inhibited the growth of subcutaneous tumors (Figure 10K). After 24 days, the mice were euthanized, and the tumor weight of the sh‐Ctrl group was significantly heavier than that of the CYP1B1 knockdown group (Figure 10L). The above results confirm the carcinogenic role of CYP1B1 in promoting gastric cancer both in vivo and in vitro.

## 4 Discussion

With the change of people's lifestyle and dietary habits, the incidence of gastric cancer is gradually increasing [28]. However, due to the lack of specific symptoms in the early stage of gastric cancer and the low acceptance of gastroscopy, most of the patients are in the advanced stage of gastric cancer when they visit the doctor [29, 30]. Distant metastasis of gastric cancer is one of the main reasons for the low five‐year survival rate of gastric cancer patients [31], and anoikis resistance confers survival conditions for gastric cancer cells to undergo distant metastasis [32], therefore, the study of ARGs is of great significance for the prognosis of gastric cancer [33, 34]. In recent years, with the development of sequencing technology, many studies have shown that driver mutations and molecular pathological subtypes affect cancer prognosis [35]. The growing research on ARGs has provided new ideas for elucidating the mechanisms of tumor metastasis. Several studies have been conducted to investigate gastric cancer‐related genes using consensus clustering, such as copper death‐related lncRNAs [36], ROS‐related genes [37], immunogenic cell death (ICD)‐related genes [38], etc., but ARGs have not been addressed yet. We used consensus clustering to make a study on the role of ARGs in gastric cancer by using consensus clustering for the first time. By typing gastric cancer based on ARGs, we can study the functional characteristics of each subtype more comprehensively and provide a theoretical basis for the study of gastric cancer pathogenesis and personalized treatment for patients.

In this study, We obtained the differentially expressed anoikis genes. By constructing a DEGs expression network and querying the miRNA expression regulation of each differentially expressed gene, we can gain a deeper understanding of the role of ARGs in gastric cancer. For example, miR‐497 is lowly expressed in anoikis‐resistant cells and can inhibit the growth and metastasis of gastric cancer cells by blocking the Wnt/β‐catenin signaling pathway and epithelial‐mesenchymal transition [39]. We performed gene set scoring for each gastric cancer sample using ssGSEA, which were finally classified into three subtypes. By WGCNA, we found that the expression patterns of these three subtypes were not identical, among which cluster2 and cluster3 had similar expression patterns and were significantly correlated with prognosis. Enrichment analysis shows significant differences in the expression patterns of the three subtypes. Studies have shown that cadmium ions induce anoikis through integrin‐related pathways [40, 41], and calcium ion endocytosis is also involved in anoikis [42, 43]; the expression patterns of cluster2 and cluster3 are similar, and top1 is enriched in intercellular adhesion, which precisely verifies the characteristics of anoikis [44]. KEGG showed that cluster1 was enriched in the chemical carcinogenesis signaling pathway, cluster2 was enriched in the extracellular matrix signaling pathway, and cluster3 was enriched in the intercellular adhesion signaling pathway, and the characteristics of anoikis were verified by cluster2 and cluster3. Reactom enrichment analysis showed that all three subtypes were enriched in the GPCR signaling pathway, which was previously found to be involved in anoikis [45, 46].

The immune analysis results showed significant differences in the abundance of activated NK cells, memory B cells, and M2 macrophages in the three subtypes. For NK cells, it has been shown that NK cells are differentially expressed in colorectal cancer subtypes classified on the basis of anoikis‐related genes [47]; for M2 macrophages, it has been shown that M2 macrophages are differentially expressed in breast cancer subtypes classified on the basis of anoikis‐related genes [48]. By calculating the correlation between the ssGSEA score and the level of immune cell infiltration, we can visualize the relationship between the differentially expressed anoikis‐related gene scores and immune cell infiltration. Among them, memory B cells were correlated in all three subtypes and overall, showing moderate correlation with anoikis. In conclusion, our study provides a new theoretical basis for the immunotherapy of gastric cancer.

Through DEGs analysis between subtypes, we found that cluster 1 and cluster 3 were both enriched in cell adhesion [49], and that altered cell adhesion to the extracellular matrix is an initiating factor for the development of anoikis [50]. By screening for differential genes among the three subtypes, we finally screened five genes (CYP1B1, EQTN, NRXN2, TBC1D3E, TCEAL5) that were differentially expressed in all three subtypes. Existing studies have shown that CYP1B1 is involved in cisplatin resistance [51] in GC and is closely related to prognosis [52] and the immune microenvironment [53], but there has been no research on the relationship between CYP1B1 and anoikis; TCEAL5 is related to prognosis in prostate cancer and cardiac signaling [54, 55], but there is no research in GC; EQTN is mainly involved in the reproductive fertilization of cattle [56] and the metabolic processes of plants [57]; NRXN2 is primarily involved in the transduction of intercellular signals in the neuropsychiatric system [58]; TBC1D3E is a membrane protein‐coding gene, and there is currently no research on it. In summary, our study provides new gene targets for the treatment of gastric cancer.

However, the role of CYP1B1 in gastric cancer anoikis has not been studied. We verified through clinical specimens of gastric cancer liver metastasis that higher expression of CYP1B1 correlates with a stronger metastatic potential of gastric cancer cells. Additionally, by constructing an anoikis model and conducting related experiments, we demonstrated that CYP1B1 can enhance the anoikis resistance of gastric cancer cells, promoting their migration and growth.

Finally, we screened 33 independent prognostic genes by univariate and multivariate COX regression and used them to construct a nomogram, and the calibration curves showed that the model was in good agreement with the actual situation. TCEAL5 is both differentially expressed among the 3 subtypes and an independent prognostic factor, so it may be a new therapeutic target.

In conclusion, for the first time, we constructed molecular subtypes of gastric cancer based on differentially expressed anoikis genes and investigated the expression pattern, enrichment pathway, immune infiltration level, and prognosis of each subtype, which provided a new perspective for the treatment of gastric cancer. However, there are some limitations in our study. All of our data came from public databases, and our conclusions should be further verified by the patient information we collected; in addition, we need some prospective studies to verify our conclusions.

## 5 Conclusion

We identified three subtypes based on ARGs and investigated the differences in the expression patterns of the three subtypes. These studies represent the most recent contribution to the field, as similar results have not been reported in previous studies and provide new ideas for the future treatment of gastric cancer.

## Author Contributions

**Chao Song:** writing – original draft. **Wenbo Liu:** writing – original draft. **Xiaoyu Wang:** software. **Xin Liu:** methodology. **Zhiran Yang:** software. **Yingying Wang:** data curation. **Qun Zhao:** formal analysis. **Yong Li:** validation. **Mingming Zhang:** supervision, writing – review and editing. **Bibo Tan:** writing – review and editing, supervision, funding acquisition, conceptualization.

## Ethics Statement

This study was approved by the Ethics Committee of the Fourth Hospital of Hebei Medical University (Project No: 2019ME0039), and Written informed consent was obtained from each patient before sample collection. All animal experiments were approved by the Ethics Committee of Hebei Medical University.

## Consent

Written informed consent was obtained from each patient before sample collection.

## Conflicts of Interest

The authors declare no conflicts of interest.

## Supporting information


Appendix S1.

## Data Availability

Public data used in this work can be obtained from the TCGA (https://portal.gdc.cancer.gov/), GeneCards (https://www.genecards.org/), Metascape (https://metascape.org), Reactome Database(http://reactom.org/), Starbase (http://starbase.sysu.edu.cn/).
