# Supplementary material for: Identification of Anoikis‐Related Genes in Gastric Cancer: Bioinformatics and Experimental Validation
Source: Cancer Med. 2025 Apr 22;14(8):e70907. doi: 10.1002/cam4.70907 (PMC12014852; doi:10.1002/cam4.70907)
Supplement: Supplementary file 1 — Appendix S1. [file CAM4-14-e70907-s001.zip › Supplement Table 5--genecard333.docx]

Supplement Table 5. The 333 anoikis genes from Genecards.

| Number | Gene |
| --- | --- |
| 1 | ABHD2 |
| 2 | ABHD4 |
| 3 | ABL1 |
| 4 | ADCY10 |
| 5 | AFP |
| 6 | AKT1 |
| 7 | AKT2 |
| 8 | AKT3 |
| 9 | ANGPTL2 |
| 10 | ANGPTL4 |
| 11 | ANKRD13C |
| 12 | ANXA5 |
| 13 | APOBEC3G |
| 14 | AR |
| 15 | ARHGEF7 |
| 16 | ATF4 |
| 17 | BAD |
| 18 | BAG1 |
| 19 | BAK1 |
| 20 | BAX |
| 21 | BCAR1 |
| 22 | BCL2 |
| 23 | BCL2L1 |
| 24 | BCL2L11 |
| 25 | BCL2L15 |
| 26 | BDNF |
| 27 | BID |
| 28 | BIRC3 |
| 29 | BIRC5 |
| 30 | BMF |
| 31 | BMP6 |
| 32 | BNIP3 |
| 33 | BNIP3L |
| 34 | BRAF |
| 35 | BRCA2 |
| 36 | BRMS1 |
| 37 | BSG |
| 38 | BST2 |
| 39 | CALR |
| 40 | CASP10 |
| 41 | CASP3 |
| 42 | CASP6 |
| 43 | CASP8 |
| 44 | CASP9 |
| 45 | CAV1 |
| 46 | CBL |
| 47 | CCAR2 |
| 48 | CCDC178 |
| 49 | CCND1 |
| 50 | CCR7 |
| 51 | CD151 |
| 52 | CD24 |
| 53 | CD36 |
| 54 | CD44 |
| 55 | CD63 |
| 56 | CDCP1 |
| 57 | CDH1 |
| 58 | CDH2 |
| 59 | CDH3 |
| 60 | CDK11A |
| 61 | CDK11B |
| 62 | CDKN1A |
| 63 | CDKN1B |
| 64 | CDKN2A |
| 65 | CDKN3 |
| 66 | CEACAM1 |
| 67 | CEACAM3 |
| 68 | CEACAM5 |
| 69 | CEACAM6 |
| 70 | CEBPB |
| 71 | CEMIP |
| 72 | CFLAR |
| 73 | CHEK2 |
| 74 | CLDN1 |
| 75 | CLU |
| 76 | COL13A1 |
| 77 | COL4A2 |
| 78 | CPEB2 |
| 79 | CPT1A |
| 80 | CRYAB |
| 81 | CSNK2A1 |
| 82 | CSPG4 |
| 83 | CTNNB1 |
| 84 | CTNND1 |
| 85 | CTTN |
| 86 | CXCL12 |
| 87 | CXCL8 |
| 88 | CXCR4 |
| 89 | CYCS |
| 90 | DAP3 |
| 91 | DAPK1 |
| 92 | DAPK2 |
| 93 | DOCK1 |
| 94 | E2F1 |
| 95 | EDA2R |
| 96 | EDIL3 |
| 97 | EEF1A1 |
| 98 | EEF2K |
| 99 | EGF |
| 100 | EGFR |
| 101 | EIF2AK3 |
| 102 | ELANE |
| 103 | ELK1 |
| 104 | EPHA2 |
| 105 | EPHB6 |
| 106 | ERBB2 |
| 107 | ETV4 |
| 108 | EZH2 |
| 109 | FADD |
| 110 | FAS |
| 111 | FASLG |
| 112 | FASN |
| 113 | FGF2 |
| 114 | FN1 |
| 115 | GDF2 |
| 116 | GLI2 |
| 117 | GLO1 |
| 118 | GLUD1 |
| 119 | GRHL2 |
| 120 | HAVCR2 |
| 121 | HGF |
| 122 | HIF1A |
| 123 | HK2 |
| 124 | HMCN1 |
| 125 | HMGA1 |
| 126 | HMOX1 |
| 127 | HRAS |
| 128 | HRC |
| 129 | HSP90B1 |
| 130 | HTRA1 |
| 131 | IFI27 |
| 132 | IGF1 |
| 133 | IGF1R |
| 134 | IL17A |
| 135 | IL1RAP |
| 136 | IL6 |
| 137 | ILK |
| 138 | INHBB |
| 139 | IQGAP1 |
| 140 | ITGA2 |
| 141 | ITGA3 |
| 142 | ITGA4 |
| 143 | ITGA5 |
| 144 | ITGA6 |
| 145 | ITGA8 |
| 146 | ITGAV |
| 147 | ITGB1 |
| 148 | ITGB3 |
| 149 | ITGB4 |
| 150 | ITPRIP |
| 151 | KDM3A |
| 152 | KDR |
| 153 | KL |
| 154 | KLF12 |
| 155 | KRAS |
| 156 | LAMA3 |
| 157 | LAMB3 |
| 158 | LAMC2 |
| 159 | LATS1 |
| 160 | LGALS1 |
| 161 | LGALS3 |
| 162 | LMO3 |
| 163 | LPAR1 |
| 164 | LRP1 |
| 165 | LTB4R2 |
| 166 | LTF |
| 167 | MAP2K1 |
| 168 | MAPK1 |
| 169 | MAPK11 |
| 170 | MAPK3 |
| 171 | MAPK8 |
| 172 | MAVS |
| 173 | MCL1 |
| 174 | MDM2 |
| 175 | MEGF11 |
| 176 | MET |
| 177 | MGAT5 |
| 178 | MIR10A |
| 179 | MIR124-1 |
| 180 | MIR141 |
| 181 | MIR145 |
| 182 | MIR1827 |
| 183 | MIR200A |
| 184 | MIR200B |
| 185 | MIR200C |
| 186 | MIR204 |
| 187 | MIR21 |
| 188 | MIR26A1 |
| 189 | MIR30B |
| 190 | MIR30C1 |
| 191 | MIR363 |
| 192 | MIR525 |
| 193 | MIR6744 |
| 194 | MMP11 |
| 195 | MMP13 |
| 196 | MMP2 |
| 197 | MMP9 |
| 198 | MNX1 |
| 199 | MSLN |
| 200 | MTA1 |
| 201 | MTDH |
| 202 | MTOR |
| 203 | MUC1 |
| 204 | MYBBP1A |
| 205 | MYC |
| 206 | MYH9 |
| 207 | MYO5A |
| 208 | NAT1 |
| 209 | NFE2L2 |
| 210 | NOTCH1 |
| 211 | NOTCH3 |
| 212 | NOX4 |
| 213 | NQO1 |
| 214 | NRAS |
| 215 | NTF3 |
| 216 | NTRK1 |
| 217 | NTRK2 |
| 218 | OLFM3 |
| 219 | PAK1 |
| 220 | PAK2 |
| 221 | PAK3 |
| 222 | PAK4 |
| 223 | PARP1 |
| 224 | PBK |
| 225 | PDCD4 |
| 226 | PDGFB |
| 227 | PDGFRB |
| 228 | PDK4 |
| 229 | PHLDA2 |
| 230 | PIK3CA |
| 231 | PIK3CB |
| 232 | PIK3CG |
| 233 | PIK3R1 |
| 234 | PIK3R2 |
| 235 | PIK3R3 |
| 236 | PIN1 |
| 237 | PLAT |
| 238 | PLAU |
| 239 | PLAUR |
| 240 | PLG |
| 241 | PLK1 |
| 242 | PPARG |
| 243 | PPP1R13B |
| 244 | PPP2CA |
| 245 | PPP2R1A |
| 246 | PPP2R2A |
| 247 | PPP2R2D |
| 248 | PPP2R5A |
| 249 | PRDX4 |
| 250 | PRKACA |
| 251 | PRKCA |
| 252 | PRKCI |
| 253 | PRKCQ |
| 254 | PRPF4B |
| 255 | PTEN |
| 256 | PTGS2 |
| 257 | PTHLH |
| 258 | PTK2 |
| 259 | PTK2B |
| 260 | PTK6 |
| 261 | PTPN1 |
| 262 | PTPN11 |
| 263 | PTRH2 |
| 264 | PYCARD |
| 265 | RAC1 |
| 266 | RAD9A |
| 267 | RAF1 |
| 268 | RANBP9 |
| 269 | RB1 |
| 270 | RBL2 |
| 271 | RELA |
| 272 | RHOA |
| 273 | RHOB |
| 274 | RHOG |
| 275 | RIPK1 |
| 276 | ROCK1 |
| 277 | RPS6KB1 |
| 278 | S100A4 |
| 279 | SATB1 |
| 280 | SDCBP |
| 281 | SEMA7A |
| 282 | SERPINA1 |
| 283 | SESN1 |
| 284 | SESN2 |
| 285 | SESN3 |
| 286 | SFN |
| 287 | SHC1 |
| 288 | SIK1 |
| 289 | SIK2 |
| 290 | SIRPA |
| 291 | SIRT1 |
| 292 | SIRT3 |
| 293 | SKP2 |
| 294 | SLC2A1 |
| 295 | SMAD4 |
| 296 | SNAI2 |
| 297 | SOD2 |
| 298 | SP1 |
| 299 | SPIB |
| 300 | SPINK1 |
| 301 | SRC |
| 302 | STAT3 |
| 303 | STK11 |
| 304 | TCF7L2 |
| 305 | TGFB1 |
| 306 | THBS1 |
| 307 | TIMP1 |
| 308 | TLE1 |
| 309 | TLN1 |
| 310 | TLR3 |
| 311 | TNFRSF10B |
| 312 | TNFRSF12A |
| 313 | TNFRSF1A |
| 314 | TNFSF10 |
| 315 | TP53 |
| 316 | TPM1 |
| 317 | TRAF2 |
| 318 | TRIM31 |
| 319 | TSG101 |
| 320 | TUBB3 |
| 321 | TWIST1 |
| 322 | UBE2C |
| 323 | VEGFA |
| 324 | VPS37A |
| 325 | VTN |
| 326 | XIAP |
| 327 | YAP1 |
| 328 | YWHAZ |
| 329 | ZBTB7A |
| 330 | ZEB1 |
| 331 | ZEB2 |
| 332 | ZNF304 |
| 333 | ZNF32 |
