# Supplementary material for: Identification of Anoikis‐Related Genes in Gastric Cancer: Bioinformatics and Experimental Validation
Source: Cancer Med. 2025 Apr 22;14(8):e70907. doi: 10.1002/cam4.70907 (PMC12014852; doi:10.1002/cam4.70907)
Supplement: Supplementary file 1 — Appendix S1. [file CAM4-14-e70907-s001.zip › Supplement.docx]

Supplement Table 1. The information of patients.

| Sample | Gender | Age(year) | T | N | M | Pathologic types |
| --- | --- | --- | --- | --- | --- | --- |
| 1 | male | 60 | 4 | 1 | 1 | adenocarcinoma |
| 2 | male | 65 | 4 | 2 | 1 | adenocarcinoma |
| 3 | female | 62 | 4 | 1 | 1 | adenocarcinoma |

Supplement Table 2. The target sequences of shRNAs.

| Gene name | Sequences (5’-3’) |
| --- | --- |
| CYP1B1#1 | GCACCGTTTTCCGCGAATT |
| CYP1B1#2 | TGGCTCACCAGTGCGATTT |

Supplement Table 3. Analysis of the differences in immune cells among the three subtypes.

| cell | *P*-value | 2-1 | 3-1 | 3-2 |
| --- | --- | --- | --- | --- |
| B.cells.naive | 0.109 | 0.122 | 0.958 | 0.205 |
| B.cells.memory | 0.025 | 0.033 | 0.961 | 0.062 |
| Plasma.cells | 0.832 | 0.945 | 0.817 | 0.963 |
| T.cells.CD8 | 0.225 | 0.874 | 0.210 | 0.493 |
| T.cells.CD4.naive | 0.407 | 0.373 | 0.789 | 0.755 |
| T.cells.CD4.memory.resting | 0.096 | 0.125 | 0.177 | 0.968 |
| T.cells.CD4.memory.activated | 0.200 | 0.244 | 0.997 | 0.273 |
| T.cells.follicular.helper | 0.789 | 0.820 | 0.828 | 0.999 |
| T.cells.regulatory..Tregs. | 0.437 | 0.433 | 0.958 | 0.595 |
| T.cells.gamma.delta | 0.502 | 0.814 | 0.831 | 0.469 |
| NK.cells.resting | 0.110 | 0.879 | 0.266 | 0.116 |
| NK.cells.activated | 0.021 | 0.695 | 0.018 | 0.166 |
| Monocytes | 0.094 | 0.130 | 0.164 | 0.981 |
| Macrophages.M0 | 0.599 | 0.584 | 0.954 | 0.757 |
| Macrophages.M1 | 0.506 | 0.548 | 0.998 | 0.578 |
| Macrophages.M2 | 0.039 | 0.041 | 0.152 | 0.791 |
| Dendritic.cells.resting | 0.606 | 0.979 | 0.725 | 0.619 |
| Dendritic.cells.activated | 0.597 | 0.997 | 0.627 | 0.693 |
| Mast.cells.resting | 0.798 | 0.782 | 0.964 | 0.907 |
| Mast.cells.activated | 0.991 | 0.998 | 0.990 | 0.997 |
| Eosinophils | 0.122 | 0.765 | 0.106 | 0.418 |
| Neutrophils | 0.426 | 0.400 | 0.704 | 0.858 |

Supplement Table 4. The primer information for qRT-PCR.

| Gene | Forword primer | Reverse primer |
| --- | --- | --- |
| CYP1B1 | TTGACTCTGGAGTGGGAGTG | TCGGTGAGTGGCGTCAATTC |
| EQTN | GCATCCACACCCTTCACTCAAGAC | ATCATCCATGACCACTGCTGTTCC |
| NRXN2 | GCACCACCCAGAACACAGATGAC | ATGGCGTAGAGGAGGATGAGGATG |
| TBC1D3E | TGGGTGGATATGCTGGGAGACTG | CCGCTGCTTGGTTCCGTATCG |
| TCEAL5 | CAGAATGCGAGGGAAAGCGAGAG | TCCTCCTGAGCCCTTGACACATC |
| β-actin | AGCGAGCATCCCCCAAAGTT | GGGCACGAAGGCTCATCATT |

**
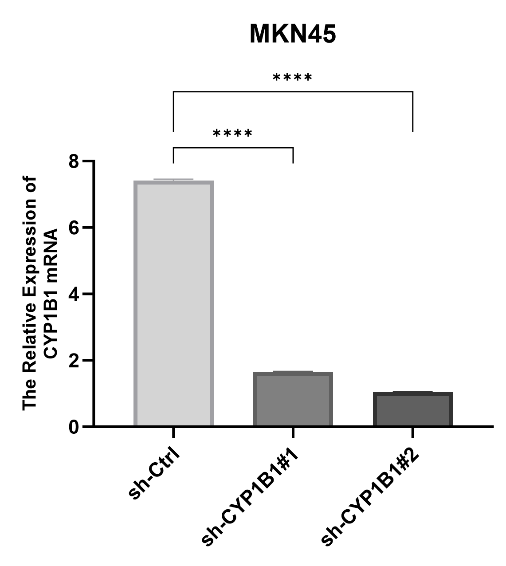
**

Supplement Figure 1. The inhibition rate of CYP1B1 in MKN45 cells were verified by qRT-PCR.


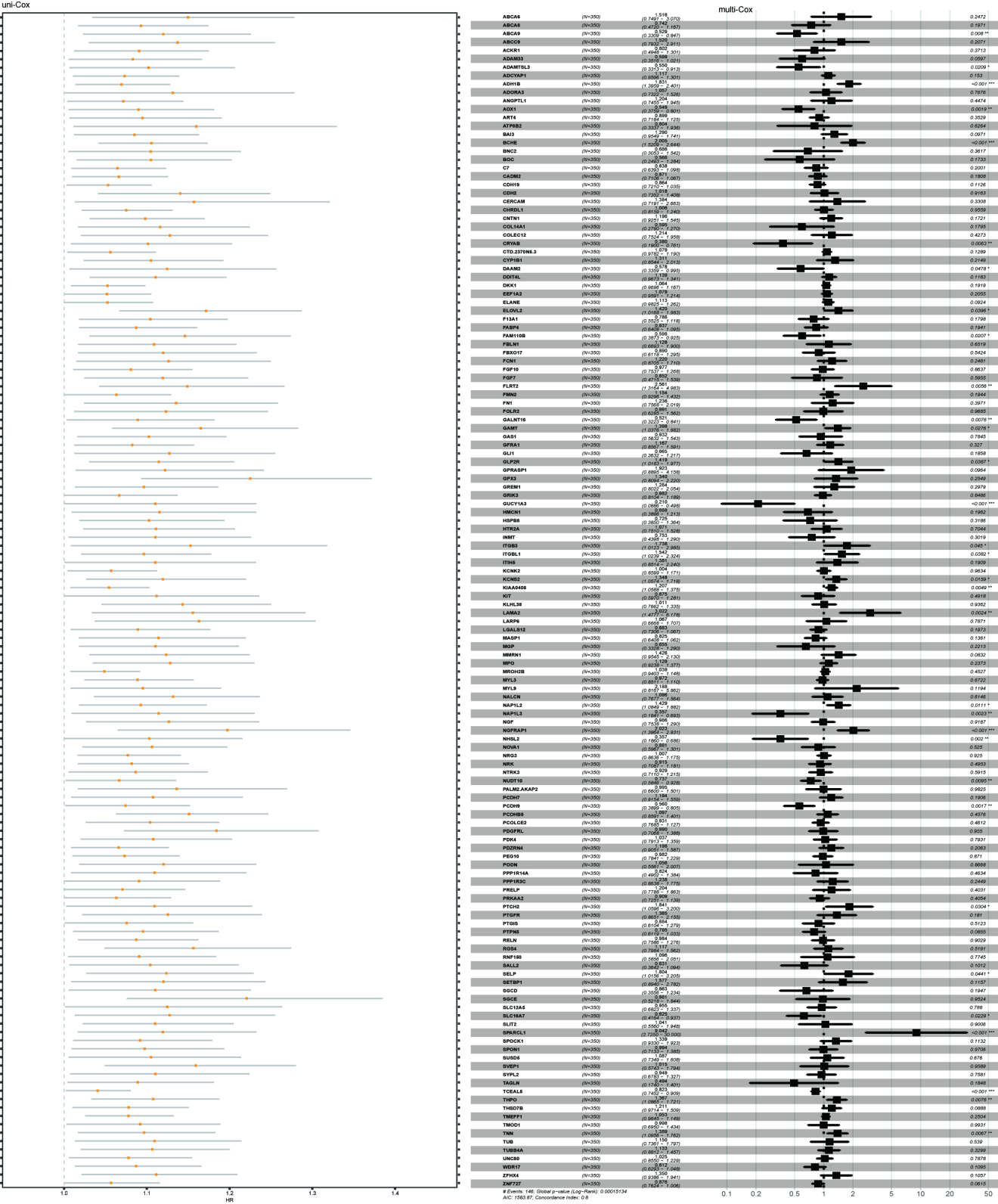


Supplement Figure 2. Univariate COX regression of 917 differentially expressed genes in at least two subtypes.


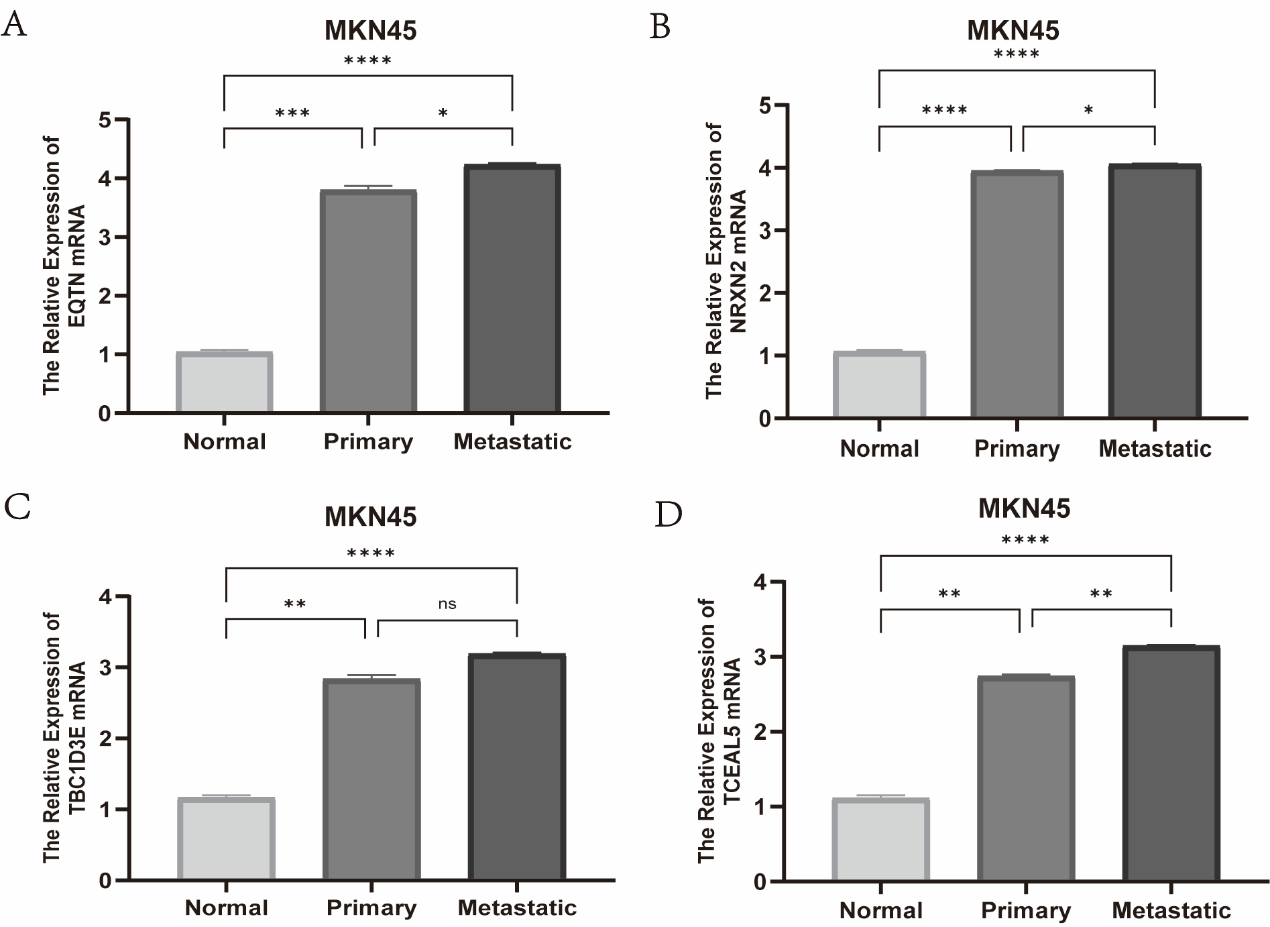


Supplement Figure 3. qRT-PCR of EQTN, NRXN2, TBC1D3E, and TCEAL5.
